# Multiplicity and molecular epidemiology of *Plasmodium vivax* and *Plasmodium falciparum* infections in East Africa

**DOI:** 10.1186/s12936-018-2337-y

**Published:** 2018-05-02

**Authors:** Daibin Zhong, Eugenia Lo, Xiaoming Wang, Delenasaw Yewhalaw, Guofa Zhou, Harrysone E. Atieli, Andrew Githeko, Elizabeth Hemming-Schroeder, Ming-Chieh Lee, Yaw Afrane, Guiyun Yan

**Affiliations:** 1 Program in Public Health, University of California at Irvine, Irvine, CA 92617 USA; 2 Department of Biological Sciences, University of North Carolina at Charlotte, Charlotte, NC 28223 USA; 3 Department of Medical Laboratory Sciences, Faculty of Health Sciences, Jimma University, Jimma, Ethiopia; 4 Tropical and Infectious Diseases Research Center, Jimma University, Jimma, Ethiopia; 5 Centre for Global Health Research, Kenya Medical Research Institute, Kisumu, Kenya; 6 Department of Medical Microbiology, College of Health Sciences, University of Ghana, Accra, Ghana

**Keywords:** Multiplicity of infection, Merozoite surface protein 1, Amplicon deep sequencing, Molecular epidemiology, *Plasmodium*, Within-host diversity

## Abstract

**Background:**

Parasite genetic diversity and multiplicity of infection (MOI) affect clinical outcomes, response to drug treatment and naturally-acquired or vaccine-induced immunity. Traditional methods often underestimate the frequency and diversity of multiclonal infections due to technical sensitivity and specificity. Next-generation sequencing techniques provide a novel opportunity to study complexity of parasite populations and molecular epidemiology.

**Methods:**

Symptomatic and asymptomatic *Plasmodium vivax* samples were collected from health centres/hospitals and schools, respectively, from 2011 to 2015 in Ethiopia. Similarly, both symptomatic and asymptomatic *Plasmodium falciparum* samples were collected, respectively, from hospitals and schools in 2005 and 2015 in Kenya. Finger-pricked blood samples were collected and dried on filter paper. Long amplicon (> 400 bp) deep sequencing of merozoite surface protein 1 (*msp1)* gene was conducted to determine multiplicity and molecular epidemiology of *P. vivax* and *P. falciparum* infections. The results were compared with those based on short amplicon (117 bp) deep sequencing.

**Results:**

A total of 139 *P. vivax* and 222 *P. falciparum* samples were pyro-sequenced for *pvmsp1* and *pfmsp1*, yielding a total of 21 *P. vivax* and 99 *P. falciparum* predominant haplotypes. The average MOI for *P. vivax* and *P. falciparum* were 2.16 and 2.68, respectively, which were significantly higher than that of microsatellite markers and short amplicon (117 bp) deep sequencing. Multiclonal infections were detected in 62.2% of the samples for *P. vivax* and 74.8% of the samples for *P. falciparum*. Four out of the five subjects with recurrent *P. vivax* malaria were found to be a relapse 44–65 days after clearance of parasites. No difference was observed in MOI among *P. vivax* patients of different symptoms, ages and genders. Similar patterns were also observed in *P. falciparum* except for one study site in Kenyan lowland areas with significantly higher MOI.

**Conclusions:**

The study used a novel method to evaluate *Plasmodium* MOI and molecular epidemiological patterns by long amplicon ultra-deep sequencing. The complexity of infections were similar among age groups, symptoms, genders, transmission settings (spatial heterogeneity), as well as over years (pre- vs. post-scale-up interventions). This study demonstrated that long amplicon deep sequencing is a useful tool to investigate multiplicity and molecular epidemiology of *Plasmodium* parasite infections.

**Electronic supplementary material:**

The online version of this article (10.1186/s12936-018-2337-y) contains supplementary material, which is available to authorized users.

## Background

Malaria is one of the most common infectious diseases and an important public health problem worldwide. In 2016, there were an estimated 216 million cases and 445,000 deaths of malaria occurred worldwide; and nearly half of the world’s population lived in 91 countries and territories are at risk of malaria transmission [1]. The majority of malaria cases and deaths (~ 90%) occur in sub-Saharan Africa. *Plasmodium falciparum* is the most prevalent malaria parasite in sub-Saharan Africa, while *Plasmodium vivax* is the most widespread human malaria with approximately 2.5 billion people at risk of infection worldwide [2]. *Plasmodium vivax* is a major cause of anaemia in an area where *P. falciparum* and *P. vivax* co-exist [3]. Relapses play an important role in the transmission of *P. vivax* in malaria endemic areas [4].With the scaling up of interventions since 2006, primarily mass distribution of insecticide-treated nets (ITNs), indoor residual spraying (IRS), and artemisinin-based combination therapy (ACT), malaria transmission has declined tremendously in the past decade [5].

The extent of genetic diversity and multiplicity of infection (MOI) is essential in understanding malaria epidemiological patterns, transmission intensity, host immune system, and parasite virulence for the development of anti-malarial vaccine as well as evaluating the impact of malaria control interventions. For example, MOI has been used for inferring disease epidemiology such as detecting parasite clearance rates subsequent to anti-malarial treatment [6] and examining the level of anti-malarial drug resistance [7, 8], the impact of transmission intensity on infection complexity [9], parasite virulence related to anti-malarial vaccine development [10, 11], and in-host ecology of malaria infections [12]. Traditional PCR-based methods, such as microsatellite [13, 14] and merozoite surface protein (*msp*) genotyping [14–17], for assessing MOI estimation can lack both sensitivity and specificity, resulting in the apparent problem of underestimating disease complexity [18–20]. Compared to genotyping methods, amplicon deep sequencing provides a rapid, robust, high-throughput approach to detect sequence variants and estimate allele frequency by sequencing a genomic region multiple times, sometimes hundreds or even thousands of times [21]. For example, ultra-deep sequencing of amplicons from the ribosomal, mitochondrion, and apicoplast encoded genes revealed a large complexity of coinfections with an unexpectedly high MOI in *Plasmodium ovale* and *Plasmodium malariae* infections in the endemic areas of Gabon [22]. Use of length polymorphic genes such as *msp2* in amplicon deep sequencing has been shown to display greater sensitivity in detecting minority clones [23].

Using *pvmsp1* short amplicon deep sequencing, Lin et al. [15] identified 67 unique haplotypes from 78 Cambodian *P. vivax* samples with an average MOI of 3.6 within each individual. Over half of the recurrent infections were detected as relapse. Compared to the standard PCR based method, next-generation sequencing revealed up to sixfold higher MOI in *Plasmodium* infections [12, 21]. This technology has unquestionably advanced our understanding of the genetics and evolution of multiclonal infection. However, in previous studies, most of amplicon deep sequencing was performed on two platforms, 454/Roche or Ion Torrent with high error rate and short reads due to technological limitation. By contrast, the Illumina MiSeq/HiSeq platform can generate reads of up to 600 bp length with lower sequencing error rate.

*Plasmodium* merozoite surface protein 1 (*msp1*) is a highly abundant and the most polymorphic antigen, which has been extensively studied in the parasite population [24–26]. *Plasmodium falciparum* has seven variable blocks that are separated either by conserved or semi-conserved regions. The variable block 2 of *pfmsp1* is the most polymorphic region of the antigen [27]. *Plasmodium vivax* has nine variable regions that are separated by 10 interspecies conserved or intraspecies conserved blocks [28]. The variable block 18, located in 42 kDa region of *pvmsp1,* has been identified to be the most polymorphic part of the antigen [11]. These polymorphic regions could be good candidate markers for multiclonal detection of *Plasmodium* infection.

The present study was designed to address the following questions: (1) how useful is amplicon ultra-deep sequencing for determining multiplicity of *Plasmodium* infection and identifying *P. vivax* relapse? (2) is there any difference in multiplicity of *Plasmodium* infection between patients of different symptoms, ages, genders, time, and transmission settings? (3) does intensified intervention since 2006 affect MOI? To address the first question, different lengths of *P. vivax* amplicons and microsatellites for MOI and relapse estimation were compared. For the second and third questions, different groups of *P. falciparum* and *P. vivax* infected patients were compared.

## Methods

### Study site and sample collection

*Plasmodium vivax* parasite samples were collected from 2011 to 2015 in two localities in Ethiopia, including Jimma (351 km away from Addis Ababa) and Asendabo (50 km away east of Jimma) located in Jimma zone of Oromia Regional state in southwestern Ethiopia. The study sites have high elevations, ranging from 1680 to 2010 m above sea level. Malaria transmission is seasonal and unstable, causing frequent epidemics in these areas [29–32]. Clinical samples of *P. vivax* from Jimma health centres or hospitals were collected during the peak transmission season (September–November) of 2014-2015, while *P. vivax* samples from Asendabo were collected from Arenado health centre and asymptomatic school children from 2011 to 2012. A total of 138 *P. vivax* samples were used in the study (Table 1). Among them, five patients from Jimma were detected with recurrent infection within 44–73 days. Both the initial and recurrent infected samples were collected from these five patients and included in this study. *Plasmodium falciparum* parasite samples were collected from symptomatic hospital patients or asymptomatic schoolchildren from 2005 to 2015 in lowland and highland areas of western Kenya (Table 1). The study sites included Kombewa and Kendu Bay lowland areas, and Iguhu and Kaimosi in highland areas. Malaria transmission in the lowland area is perennial and hyperendemic, while transmission intensity in the highland is mesoendemic [33–37]. All blood samples were obtained by finger-pricking and dried on filter paper as described previously [34, 38–41].

**Table 1:** Sample collection of *Plasmodium vivax* in Ethiopia and *Plasmodium falciparum* in Kenya

Country	Species	Location	Elevation (m)	Year	Endemicity^a^ [refer]	Symptoms	n	Reads^b^ (> 1 k)
Ethiopia	*P. vivax*	Jimma	1680–1700	2014–2015	2.4^PCR^ [31]	Sym	67	65
		Asendabo	1710–2010	2011–2012	32.4^MIC^ [30]	Sym	48	47
		Asendabo	1710–2010	2011–2012	32.4^MIC^ [30]	Asym	23	23
Kenya	*P. falciparum*	Iguhu	1420–1600	2005	36.5^MIC^ [36]	Sym	29	29
		Iguhu	1420–1600	2005	36.5^MIC^ [36]	Asym	29	28
		Iguhu	1420–1600	2015	15.0^MIC^ [37]	Asym	22	22
		Kaimosi	1600–1700	2015	11.0^MIC^ [35]	Asym	29	27
		Kendu Bay	1100–1200	2015	59.3^PCR^ [34]	Asym	29	29
		Kombewa	1150–1250	2005	49.4^MIC^ [36]	Sym	29	29
		Kombewa	1150–1250	2005	49.4^MIC^ [36]	Asym	29	29
		Kombewa	1150–1250	2015	43.5^MIC^ [37]	Asym	29	29

*Sym* symptomatic; *Asym* asymptomatic; *n* number of samples

^a^Community asymptomatic parasite prevalence by microscope examination (MIC) or PCR detection (PCR)

^b^Number of samples with total joined sequence reads more than 1000

### PCR amplification and deep sequencing of *pvmsp1* and *pfmsp1*

**Fig. 1:**
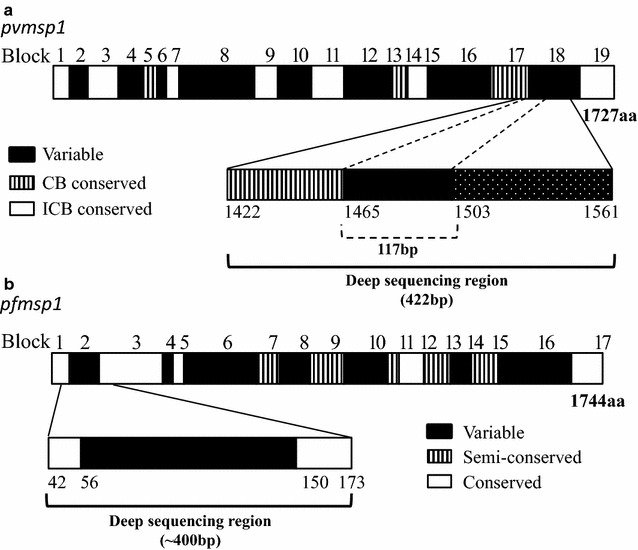
Schematic diagrams of the *msp1* protein and amplicon deep sequencing region. **a** *pvmsp1* blocks represent interspecies conserved blocks (ICBs)(white), conserved blocks (CBs) (hatched) and variable regions (black) according to del Portillo et al. [28]; **b** *pfmsp1* blocks represent conserved regions (white), semi-conserved regions (hatched) and variable regions (black) according to Tanabe et al. [27]

DNA was extracted from dried blood spots on filter paper by the Saponin/Chelex method [42] and genomic DNA was eluted in a total volume of 200 μl TE buffer. Molecular identification of *P. vivax* and *P. falciparum* for each sample was assessed by nested PCR amplification with species-specific primers designed on the small subunit ribosomal ribonucleic acid (18S rRNA) genes [43, 44]. PCR products for Illumina Hiseq sequencing were prepared by two-step PCR approach using primers specific for highly variable region of *pvmsp1* [28] and *pfmsp1* [27] genes (Fig. 1). The first PCR used appended 5′ end forward and reverse target gene specific primers, while the second PCR used universal primer with barcode primers appended 5′ end (see Additional file 1). The length of PCR products are 463 bp for *P. vivax* (refer to AF435593), and 395 bp for *P. falciparum* (refer to NC_004330).

PCR amplification of each sample was conducted in a 20 μl reaction mixture containing 2 μl of genomic DNA, 4 μl of 5 × PCR buffer, 1 unit of high fidelity PrimeSTAR^®^ GXL DNA Polymerase (Takara Bio USA, Inc., Mountain View, CA), and 10 pmol of each primer. The laboratory strains *P. vivax* Pakchong (MRA-342G) and *P. falciparum* 3D7 (MRA-102G) were also included as control. Amplification reactions were performed with an initial denaturation at 94 °C for 3 min, followed by 35 cycles at 94 °C for 30 s, 55 °C for 30 s and 72 °C for 60 s, with a final 6-min extension at 72 °C. Ten samples from each species were amplified in duplicate with unique barcode for confirmation of amplicon. Amplicons were cleaned and normalized to 1–2 ng/μl concentration using the SequalPrep Normalization Plate Kit (Life Technologies, Carlsbad, California). HiSeq Rapid SBS Kit v2 (with reads up to 2 × 250 bp) was used for library preparation. Multiple samples were pooled and sequenced on the Illumina Hiseq 2500 (384-well plate with dual indexing, UCI Genomics High-Throughput Facility).

### Haplotype determination from deep sequencing

Haplotypes of *pvmsp1* and *pfmsp1* variants were determined by SeekDeep software developed by Bailey lab at University of Massachusetts Amherst (http://baileylab.umassmed.edu/seekdeep). This software uses a clustering method to construct the most likely haplotypes within a patient while removing false haplotypes due to PCR or sequencing error [15]. Before running data on SeekDeep software, all paired-end reads were merged using Fastq-join software with the parameters: Number of percent maximum difference = 8, Number of minimum overlap = 30. Joined reads of each sample were grouped into different clusters after trimming of barcodes, tags, and primers. For each sample, haplotype clusters were determined by within-host reads cutoff frequency at 2.0%. EstimateS (v 9.1.0) program [45] was used to infer estimates of allelic richness. Sample-based rarefaction (haplotype accumulation) curves were plotted with 95% confidence intervals. The input matrix used *msp1* haplotype abundance or incidence data for a set of related samples. Relapse or reinfection of *P. vivax* was classified based on previously published method [15].

### Comparison of amplicon deep sequences of different length and microsatellite genotyping

To compare longer and short sequencing fragment in MOI determination, 117 bp fragment of *pvmsp1* was extracted, which had the same size as the ones previously used for amplicon deep sequencing [15]. MOI was evaluated using the same procedure as describe above. Our previous microsatellite genotyping data of *P. vivax* [39] were also included for comparison in the study. For *P. falciparum*, the shorter amplicon was not able to be extracted for comparison, due to large length difference and extensively polymorphism in the amplicon region. Analysis of variance (ANOVA) and mean comparisons were performed using the JMP statistical software package (JMP 12.2.0; SAS Institute, Cary, NC). Mean MOI was compared using the Tukey–Kramer HSD test (alpha = 0.05) or Student’s t test.

### Sequence variation analysis and haplotype relationship within multiple infections

MAFFT v7 online version (https://mafft.cbrc.jp/alignment/server/) was used to align DNA sequences [46]. Bioedit v7 was used to calculate sequences identity [47]. Analysis of haplotype and nucleotide diversity was performed by using DnaSP v5 [48]. The Nei’s unbiased expected heterozygosity (He) was calculated as a measure of overall genetic diversity for each genotype method [49]. Analysis of Molecular Variance (AMOVA) was conducted by GenAlEx 6.5 to estimate sequences variation within- and between infections [50]. The MEGA v7 was used to create a UPGMA phylogenetic tree [51]. The tree was annotated using the online tool iTOL (interactive Tree of Life) v3 program (http://itol.embl.de/index.shtml) [52]. The PopART v1.7 software was used to construct a minimum spanning haplotype network between haplotypes [53].

## Results

### Sequence reads and haplotype determination

A total of 384 PCR reactions (362 samples and 2 controls as well as 20 replicate PCR reactions) were successfully amplified and sequenced, resulting in 166 M total reads, of which 120 M reads (74%) passed filter, including 100 M with Qscores > 30, with an exception of three *P. vivax* samples and two *P. falciparum* samples with less than 1000 reads that were excluded from the analyses. The haplotype clustering threshold was determined by a subset of samples with replicate PCR reactions. These samples were analysed separately by SeekDeep and the results were compared among replicates as well as with a single clone lab strain (Pakchong or 3D7) as a positive control sample. A threshold cutoff frequency of 2.0% was determined to provide identical results for minor clonal calling between replicates, instead of 0.5% default threshold cut-off [54].

For the 135 *P. vivax* samples, a total of 11,576,219 joined reads were obtained by the fast-join program, of which 4,657,238 (36.1%) were successfully clustered by SeekDeep with an average of 34,498 reads per sample at within-host cluster frequency > 2.0%. The *pvmsp1* amplicon generated identical 422 bp fragment with 88 haplotypes. Among them, 21 *P. vivax* predominant haplotypes (the clone had the highest frequency within infection) were identified (GenBank acc. MG657437–MG657457, Additional file 2). NCBI nucleotide BLAST search identified that 10 out of the 21 unique haplotypes had a perfect match with sequences from GenBank and > 99% sequence similarity for the others against distinct sequences from GenBank (see Additional file 3).

For the 222 *P. falciparum* samples, a total of 42,832,457 merged reads were generated, of which 23,187,282 (52.5%) joined reads were clustered with an average of 104,447 reads per sample at within-host cluster frequency > 2.0%. The length of *pfmsp1* amplicons varied from 239 to 410 bp with 307 unique haplotypes. Among them, 99 *P. falciparum* predominant haplotypes were identified (GenBank acc. MG675458-MG675556, Additional file 4). NCBI nucleotide BLAST search identified that 25 out of the 99 unique haplotypes had a perfect match with sequences from GenBank and a range of 84.4–99.7% sequence similarity for the others against distinct sequences from GenBank (see Additional file 5).

### Haplotype diversity and population frequency distribution

**Fig. 2:**
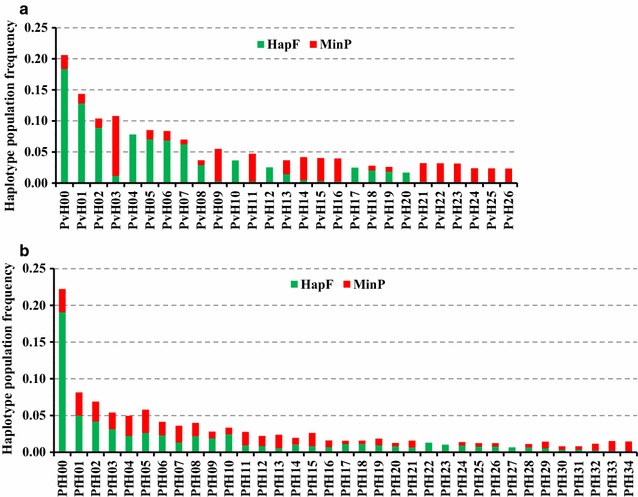
Frequency of unique *msp1* haplotypes within the study population. Only haplotypes that appeared in more than 2 samples are shown. The green bar (HapF) represent the frequency of haplotype and the red portions of the columns (MinP) represent the proportion that occurred as a minority variant (existing at 0.5–20% frequency within the individual sample). **a** *P. vivax* population; **b** *P. falciparum* population

**Fig. 3:**
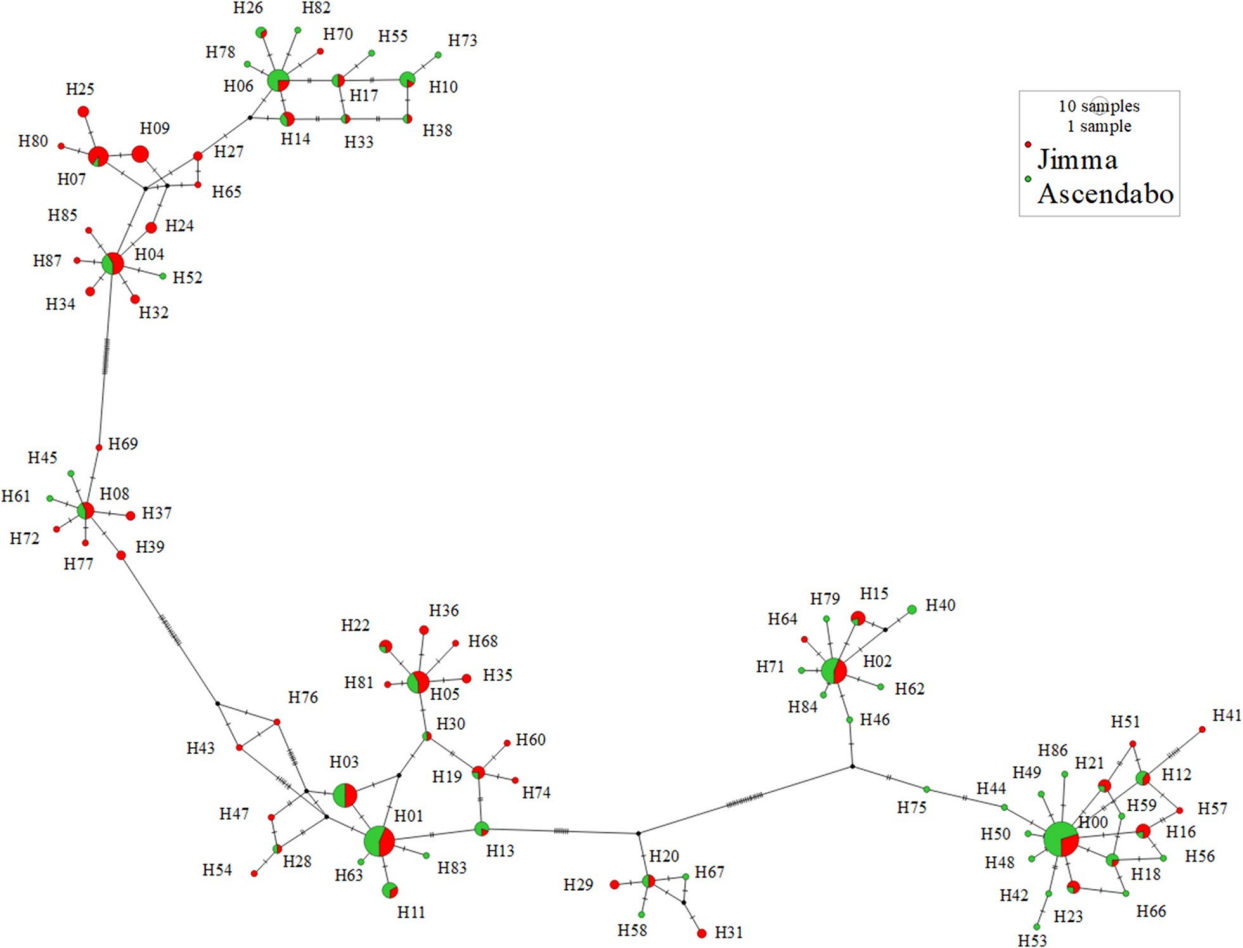
Minimum spanning networks of *pvmsp1* haplotypes showing all variants detected (H00–H87). Frequency of haplotypes is indicated by circle size; circle fill color indicates location; numbers in bracket near the connection lines indicate the number of mutations

**Fig. 4:**
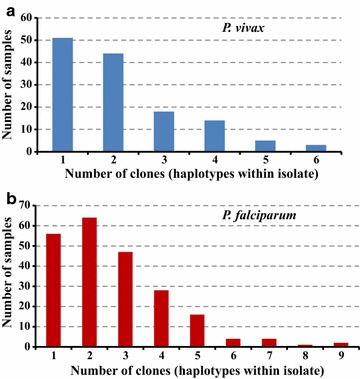
Multiplicity of infection in *P. vivax* and *P. falciparum* populations. **a** blue bar chart shows number of samples in single and polyclonal infection of *P. vivax*; **b** red bar chart shows number of samples in single and polyclonal infection of *P. falciparum*

**Fig. 5:**
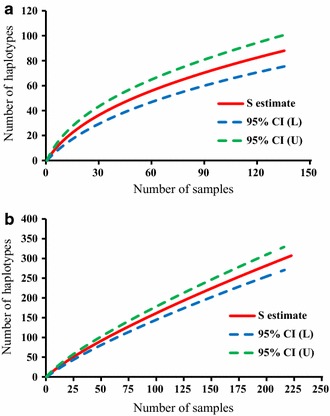
Sample-based rarefaction curves for haplotype richness. **a** *P. vivax*; **b** *P. falciparum*. The graphs show rarefaction curve (S estimate, in red solid line) with 95% confidence intervals (CI, in dashed lines). *L* lower limit, *U* upper limit

The 88 unique *pvmsp1* haplotypes exhibited 73 variable (polymorphic) sites, including 12 singleton variable sites. The average haplotype diversity (Hd) and nucleotide diversity (Pi) were 0.969 and 0.055, respectively. All of the 88 haplotypes could be successfully translated into completed amino acid sequences, resulting into 52 distinct amino acid haplotypes. Eight common nucleotide haplotypes each appeared in at least 10 samples (Fig. 2a, Additional file 2), while nearly half (47/88) of haplotypes appeared in only 1 individual sample with within-host frequency ranging from 3.4 to 100%. Approximately 70% (62/88) of the identified haplotypes were detected only as minority variants (within-host frequency < 20%). Some of these minority variants were detected from multiple samples (H09) and multiple locations (H11 and H14–H16). Other minority variants detected formed part of the mutational path (e.g. H43 and H76) between the more common variants, as depicted in a minimum spanning network based on sequence relatedness, adding support that they are true haplotypes and not a result of PCR or sequencing error (Fig. 3). Polyclonal infections were detected in 62.2% (84/135) of samples, ranging from 2 to 6 clones per sample (Fig. 4a). The Nei’s unbiased expected heterozygosity at this locus was He = 0.92, representing an average 92% probability for 2 parasite clones harboring different *pvmsp1* haplotypes in the population. Estimates of allelic richness in all the 135 *P. vivax* samples indicated that there was no clear plateau in accumulation curves (Fig. 5a), suggesting more haplotypes would be expected to occur from increased samples.

For the 307 unique *pfmsp1* haplotypes, a total of 20 amplicons with various fragment length were identified, of which the three amplicon sizes 266, 311, and 338 bp appeared in at least 10% of haplotypes (see Additional file 4). All 307 haplotypes could be successfully translated into completed amino acid sequences, resulting in 262 distinct amino acid haplotypes. Eight common nucleotide haplotypes each appeared in at least 10 samples (Fig. 2b, Additional file 4), while nearly 80% (243/307) of haplotypes appeared in only 1 individual sample with within-host frequency ranging from 3.4 to 100%. Approximately half (161/307) of the identified haplotypes were detected only as minority variants. Some of these minority variants were detected from multiple samples and multiple locations. NCBI nucleotide BLAST search identified that 9 out of the 161 minority haplotypes had a perfect match with sequences from GenBank and a range of 91.5–99.7% sequence similarity for the others against distinct sequences from GenBank (Additional file 5). Polyclonal infections were detected in 74.8% (166/222) samples, ranged from 2 to 9 clones per sample (Fig. 4b). The Nei’s unbiased expected heterozygosity at this locus was He = 0.95, indicating an average 95% probability to get 2 parasites clones with different *pvmsp1* haplotypes from the population. Similar to *P. vivax* samples, there was no clear asymptote in accumulation curves from the estimates of allelic richness in all the 222 *P. falciparum* samples (Fig. 5b), suggesting more haplotypes might be found from increased samples.

### Comparison of three methods for MOI and relapse determination in *P. vivax* infection

**Fig. 6:**
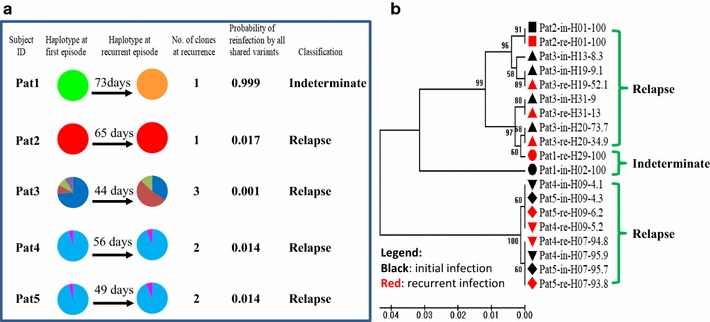
Deep sequencing identification of *P. vivax* relapse. **a** Pie chart represents haplotype composition changes between initial and recurrent infections. Each color represents a unique haplotype. Probability of reinfection calculated by population frequency of all shard haplotypes; Classification: *P* < 10%, relapse; otherwise, Indeterminate (Lin et al. [15]). **b** Phylogenetic analysis of relapse. Branch labels in black markers are initial infected haplotype; labels in red markers are recurrent infections. Label used patient number followed by haplotype name and frequency of the haplotype within host infection

To examine whether longer amplicon fragment is better for MOI detection and relapse identification in *P. vivax*, the amplicon length was extended from 117 bp [15] to 422 bp for deep sequencing. The results indicated that a longer amplicon had significantly higher MOI (2.16) than that of short amplicon deep sequencing (1.64), and microsatellite markers (1.07) (F_2,324_ = 27.1, *P* < 0.0001) (Table 2). Multiclonal infections were detected in 62.2% samples by long fragment deep sequencing, while 45.9% by short fragment deep sequencing and only 5.2% by microsatellite markers. Likewise, number of haplotypes (allele) is higher with a long fragment than that of a short fragment and microsatellites (Table 2). Among the five patients who were detected with recurrent *P. vivax* infection, four of them were identified as relapse infection and one as indeterminate by long amplicon deep sequencing (Fig. 6a). Phylogenetic analysis of haplotypes in the relapse patients indicated that haplotypes were identical between initial and relapse infections, except patient 3 (Pat3) who had an extra minor clone in the initial infections (Pat3-in-H013-8.3) (Fig. 6b). Patient 1 had a different haplotype between initial (Pat1-in-H002-100) and recurrent infections (Pat1-re-H029-100), indicating a new infection or indeterminate.

**Table 2:** Comparison of three methods for determination of multiplicity of infection (MOI) of *P. vivax*: long fragment (422 bp) of *pvmsp1* amplicon deep sequencing, short fragment (117 bp) of *pvmsp1* amplicon deep sequencing, and microsatellite marker genotyping

	Amplicon deep sequencing Long fragment (422 bp)	Amplicon deep sequencing Short fragment (117 bp)	Microsatellite markers^a^
Number of subject	135	135	58
Median MOI	2	1	1
Mean MOI^a^	2.16^a^	1.64^b^	1.07^c^
Max MOI	6	4	3
No. polyclonal	84	62	3
% polyclonal	62.2	45.9	5.2
No. alleles	88	29	24
Heterozygosity (He)	0.92	0.84	0.77

Significant differences were detected in mean MOI among the three methods as indicated by the superscripts (Tukey–Kramer HSD test, *P* < 0.05)

He: Expected heterozygosity corrected for sample size

^a^Refer to Lo et al. (2015) [39]

### Multiplicity of *P. vivax* infections in different patient groups

**Fig. 7:**
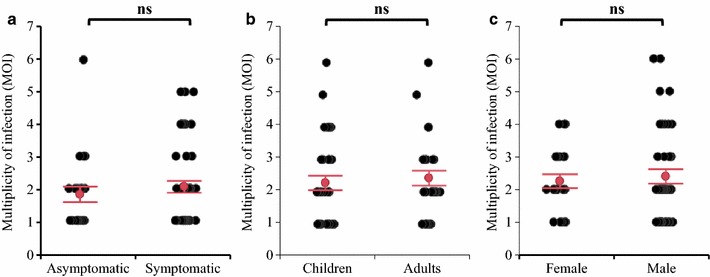
Scatter dot plot representation of multiplicity of *Plasmodium vivax* infections. MOI estimates were based on the longer amplicon deep sequencing in patients with different symptoms (**a**), ages (**b**), and genders (**c**) in Ethiopia. *Error bars* 95% confidence interval for mean, *ns*: not significant different by ANOVA analysis at *P* < 0.05 level

For the *P. vivax* samples collected from Asendabo in 2011–2012, the percentage of polyclonal infection was 52.2% for asymptomatic and 55.3% for symptomatic patients, respectively. The average MOI in symptomatic patients (n = 47) was 2.06 ± 018, which was slightly higher than that of the asymptomatic individuals (MOI = 1.83 ± 0.26, n = 23), though the difference was not significant (ANOVA, F_1,68_ = 0.570, *P *= 0.453) (Fig. 7a). Among the symptomatic *P. vivax* samples collected from Jimma in 2014–2015, there was no significant difference in MOI between children and adults (ANOVA, F_1,63_ = 0.212, *P *= 0.647) as well as between females and males (ANOVA, F_1,63_ = 0.185, *P *= 0.668) (Fig. 7b and 7c). The percentage of polyclonal infection ranged from 65.7 to 76.7%, which was slightly higher than that of patients from Asendabo.

### Multiplicity of *P. falciparum* infections in different patient groups

**Fig. 8:**
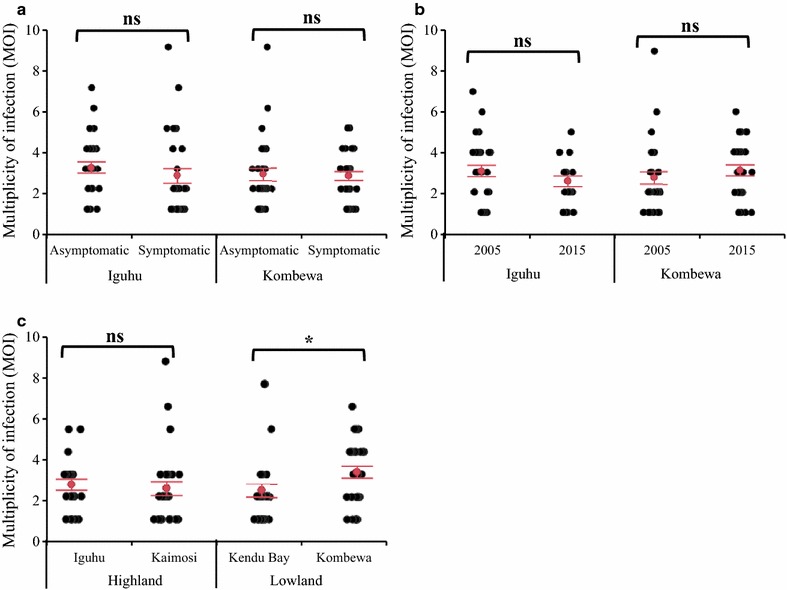
Scatter dot plot representation of multiplicity of *Plasmodium falciparum* infections. MOI estimates were based on the longer amplicon deep sequencing in patients with different symptoms (**a**), Years (**b**), and locations (**c**) in Kenya. Error bars: 95% confidence interval for mean. * indicate significant difference at *P* < 0.05 level; ns: not significant different by ANOVA analysis at *P* < 0.05 level

No significant difference was observed between asymptomatic and symptomatic *P. falciparum* samples from Kombewa (ANOVA F_1,56_ = 0.032, *P *= 0.860) and Iguhu (ANOVA F_1,55_ = 0.728, *P *= 0.397) collected in 2005 (Fig. 8a). Similarly, no difference of MOI was observed from 2005 to 2015 in both Kombewa, the lowland area (ANOVA, F_1,56_ = 0.832, *P *= 0.366) and Iguhu, the western Kenya highland area (ANOVA, F_1,48_ = 1.647, *P *= 0.206) (Fig. 8b). To examine MOI difference in different transmission settings, the asymptomatic samples collected from western Kenya highland (Iguhu and Kaimosi) and lowland (Kombewa and Kendu Bay) in 2015 were compared. Overall, no significantly difference of MOI was observed among the four study sites (ANOVA, F_3,103_ = 1.781, *P *= 0.155). However, the MOI detected in Kombewa (lowland) was 3.10, which was relatively higher than that of the other three sites (2.54 for Iguhu, 2.37 for Kaimosi, and 2.27 for Kendu Bay) and reached significant difference level at *P* < 0.05 between Kombewa and Kendu Bay when the difference was determined by a Student’s t test (Fig. 8c).

## Discussion

Multiplicity of infection (MOI), also termed complexity of infection (COI) is defined as the number of different parasite strains co-infecting a single host. MOI can be a useful indicator of immune status and transmission level. Traditionally, MOI was assessed by PCR genotyping of antigen protein genes (*msp1, msp2,* and *glurp*) and microsatellite markers, which were regarded as the gold standard because of their high polymorphism [22]. However, these methods were unable to distinguish sequence variation among parasite strains and detect minority clones within a host. By using next-generation amplicon deep sequencing, the minority clone could be detected as low as 0.5% within-host infection frequency [6, 15]. In the study, the Illumina HiSeq platform combined with Rapid SBS Kit v2 generated reads up to 500 bp with high coverage depth (~ 35 k × for *P. vivax* and ~ 100 k × for *P. falciparum*). Compared to a previous study by Lin et al. that employed a 117 bp-fragment of *pvmsp1* short amplicon deep sequencing [15], longer amplicon sequencing, by capturing a greater number of polymorphisms, revealed a higher MOI and improved power to detect multiclonal infections. Interestingly, using microsatellite markers with the same parasite population, multiclonal infections were detected only in 5.2% of the samples with an average MOI of 1.07 [39], significantly lower than that estimated by longer amplicon sequencing (a mean of 2.16 MOI). One possible reason might be the missed genotyping of polyclonal infections in some of the tested samples with microsatellite analysis. Such contrast suggested that transmission intensity may not be low. Together with high relapse as identified in the present study, there could be a much larger *P. vivax* reservoir that sustains continual transmission and makes elimination challenging.

The complexity of infection has been suggested to be associated with ages and symptoms in *Plasmodium* infections [55–58]. However, in this study, no significant difference was found in *P. vivax* MOI between the symptomatic and asymptomatic infections, adults and children, as well as between male and female groups. Similar patterns were also reported in other studies [59, 60]. In western Kenya, no notable difference was detected in the multiplicity of *P. falciparum* among asymptomatic school children in low transmission areas (highland) and in high transmission areas (lowland) over 10 years. However, in the high transmission areas (lowland), significantly difference in MOI was detected between locations (Kombewa vs Kendu Bay). The temporal changes in complexity of *P. falciparum* infections could be varied by transmission intensity and our findings indicated that multiclonal parasite genotypes could have remained steady over time in high transmission areas. Several researchers have reported correlations between clinical symptoms and higher MOI [60–68] while others did not find any associations [69–71]. Some studies reported that a reduced risk of clinical malaria was associated with multiclonal infections [72–74], while other studies reported that mono-infections and very common genotypes are more likely to develop severe malaria than multiclonal infections [70, 75]. A positive association between the proportion of polyclonal infections and parasite prevalence has been observed in parasite populations from Indonesia [76] and Papua New Guinea [77], while in other studies, no association or negative correlation was found between the rate of polyclonal infections and parasite prevalence [77, 78]. In Ethiopia, reported malaria cases were respectively 2.6 million and 2.2 million in 2011 and 2015, however, proportion of *P. falciparum* increased by 5% from 2011 to 2015 (Zhou unpublished data), indicating a relative weak reduction in transmission. In our study areas in Kenya, malaria parasite prevalence in school children in the lowland increased from 40 to 45% from 2011 to 2015 while it decreased from 16% in 2011 to 13% in 2015 in the highlands, results also indicated insignificant changes in transmission in the areas [37].

Long amplicon deep sequencing of *msp1* offers a sensitive tool to detect relapse, defines multiclonal-infected samples, and elucidates within-host genetic diversity and parasite relationships among infections [12, 79–82]. In the present study, a close genetic relationship was found among *P. vivax* clones within-hosts, which explained less than ~ 30% of the total variance when compared to between-host infections (Table 3). This result suggested that *pvmsp1* haplotypes were more genetically similar within than between hosts. Similar pattern was also observed in *P. falciparum* [83]. The close relatedness among the parasite strains within a host could be a result of frequent recombination and/or selection for drug resistant strains. Further investigation is needed to understand the mechanism generating within-host diversity.

**Table 3:** Analysis of molecular variance (AMOVA) of *P. vivax* infections using *pvmsp1* deep sequencing

Source of variation	*df*	SS	MS	Est. variance	Variation (%)	*P*
Among individuals	83	2276.02	27.42	8.36	70.7	< 0.001
Within individuals	157	543.45	3.46	3.46	29.3	
Total	240	2819.47		11.83	100	

In the study, using long amplicon deep sequencing of high polymorphic makers, *pvmsp1* and *pfmsp1*, minority clones were able to be detected in multiclonal infections. However, there are also a few limitations in the study: (1) only a small polymorphic genomic region is amplified, not covered whole genome variants; (2) the threshold for haplotype cluster calls needs to be determined by empirical methods in each study, due to various sequencing error rates in different sequencing platforms and computational strategies; (3) the PCR slippage might be present in early PCR cycle at the microsatellite repeat unit of length polymorphic *pfmsp1* marker, which resulted in increased frequency of minority clones; (4) there was only a subset of samples with replicate PCR. In order to exclude PCR or sequencing errors, it is better to perform experiments in duplicate of all the samples and use appropriate controls in each study to help determine that no false calls are being made; (5) the low percentage of reads was clustered in our clinical samples compared to laboratory strain (> 99%), suggesting DNA template quality is important. This can be improved by removal of host DNA using an enzyme-based DNA degradation method that selectively digests and depletes human DNA contamination from malaria clinical samples [84]. Another limitation is the lack of a mixed infection positive control, especially for *pfmsp1* with the different product fragment lengths.

## Conclusions

Long-amplicon deep sequencing is a powerful, high-throughput, sensitive method in measuring *Plasmodium* MOI and within-host diversity. Multiclonal infections were common among age groups, symptoms, genders, transmission settings, as well as over years (pre- vs. post-scale-up interventions) in *P. vivax* and *P. falciparum* infections. The study demonstrated that long amplicon deep sequencing is a useful tool to investigate multiplicity and molecular epidemiology of *Plasmodium* parasite infections.

## Additional files

**Additional file 1.** List of two-step PCR primers for amplicon deep sequencing in *P. falciparum pfmsp1* and *P. vivax pvmsp1* genes.

**Additional file 2.** Amplicon deep sequencing data of 135 *P. vivax* samples.

**Additional file 3.** Population frequency, sample counts, and GenBank blast results of the 88 haplotypes identified in the 135 *P. vivax* samples.

**Additional file 4.** Amplicon deep sequencing data of 222 *P. falciparum* samples.

**Additional file 5.** Population frequency, sample counts, and GenBank blast result of the 307 haplotypes identified in the 222 *P. falciparum* samples.
