# Supplementary material for: Multiplicity and molecular epidemiology of Plasmodium vivax and Plasmodium falciparum infections in East Africa
Source: Malar J. 2018 May 2;17:185. doi: 10.1186/s12936-018-2337-y (PMC5932820; doi:10.1186/s12936-018-2337-y)
Supplement: Supplementary file 1 — Additional file 1. List of two-step PCR primers for amplicon deep sequencing in P. falciparum pfmsp1 and P. vivax pvmsp1 genes. [file 12936_2018_2337_MOESM1_ESM.docx]

Additional file 1: List of two-step PCR primers for amplicon deep sequencing in *P. falciparum pfmsp1* *and P. vivax pvmsp1* genes.

| Primer | Sequence | Product length (bp) |
| --- | --- | --- |
| **First step PCR:** |  |  |
| *pvmsp1* Forward primer | TCGTCGGCAGCGTCAGATGTGTATAAGAGACAG N[0-3] ACCCATACAAGCTGCTCGAC | 463 |
| *pvmsp1* Reverse primer | GTCTCGTGGGCTCGGAGATGTGTATAAGAGACAG N[0-3] TCCTCCAACTTCTCATCCATC |  |
|  |  |  |
| *pfmsp1* Forward primer | TCGTCGGCAGCGTCAGATGTGTATAAGAGACAG N[0-3] GAAGCTTTAGAAGATGCAGTATTGA | 395 |
| *pfmsp1* Reverse primer | GTCTCGTGGGCTCGGAGATGTGTATAAGAGACAG N[0-3] TCAAAGAGTTCGGGATATTTGAG |  |
|  |  |  |
| **Second step PCR:** |  |  |
| Forward primer | AATGATACGGCGACCACCGAGATCTACAC[NNNNNNNN, barcode] TCGTCGGCAGCGTC |  |
| Reverse primer | CAAGCAGAAGACGGCATACGAGAT[NNNNNNNN, barcode] GTCTCGTGGGCTCGG |  |

N[0-3](N, NN, and NNN) are mixed sequence bases added to introduce sequence complexity and to improve their coverage. The underlined nucleotides ware gene specific primers. The length of PCR product is referred to *P. vivax* strain *Sal-1* (AF435593) and *P. falciparum* strain *3D7* (NC_004330) excluding primer adapters.
